# Molecular evidence of IGFBP-3 dependent and independent VD3 action and its nonlinear response on IGFBP-3 induction in prostate cancer cells

**DOI:** 10.1186/s12885-020-07310-5

**Published:** 2020-08-24

**Authors:** Ko Igarashi, Yoshihiro Yui, Kenta Watanabe, Jun Kumai, Yasuko Nishizawa, Chisato Miyaura, Masaki Inada, Satoru Sasagawa

**Affiliations:** 1 Molecular Biology Laboratory, Research Institute, Nozaki Tokushukai Hospital, Tanigawa 2-10-50, Daito, Osaka, 574-0074 Japan; 2 Department of Biotechnology and Life Science, Faculty of Engineering, Tokyo University of Agriculture and Technology, Nakamachi 2-24-16, Koganei, Tokyo Japan; 3 Sarcoma Treatment Laboratory, Research Institute, Nozaki Tokushukai Hospital, Tanigawa 2-10-50, Daito, Osaka, Japan; 4 Department of Orthopedics, School of Medicine, Toyama University, Sugitani, Toyama, 2630 Japan; 5 Pathology Laboratory, Research Institute, Nozaki Tokushukai Hospital, Tanigawa 2-10-50, Daito, Osaka, Japan

**Keywords:** Vitamin D, Nonlinear IGFBP-3 induction, Bcl-2 suppression, Prostate cancer treatment

## Abstract

**Background:**

Clinical trials have been conducted to clarify the beneficial effects of VD3 (1α,25-dihydroxy vitamin D3, also known as calcitriol) treatment in prostate cancer. However, the molecular mechanisms underlying these effects are not fully understood. Recent studies on IGFBP-3 have indicated its intracellular functions in cell growth and apoptosis. The aim of this study was to confirm the benefits of low-dose VD3 treatment and clarify the molecular mechanisms underlying these beneficial effects in prostate cancer cells.

**Methods:**

The molecular effects of simultaneous treatment of LNCaP cells and their genetically modified cell lines with low concentration of docetaxel and VD3 were biologically and biochemically analyzed. To further determine the effects of VD3 treatment on IGFBP-3 induction system, cells were temporarily treated with VD3 in combination with a transcriptional inhibitor or protein synthesis inhibitor. Bcl-2 protein and its mRNA behavior were also observed in Igfbp-3 expression-modified LNCaP cells to determine the involvement of IGFBP-3 in the suppression of Bcl-2 by VD3 treatment.

**Results:**

Changes in IGFBP-3 expression levels in LNCaP cells indicated that it mediated the inhibition of cell growth induced by VD3 treatment. IGFBP-3 was also found to be a mediator of the enhanced cytotoxicity of prostate cancer cells to VD3 in combination with the anti-cancer drug. We further identified the distinct property of the IGFBP-3 induction system, wherein temporal VD3 stimulation-induced prolonged IGFBP-3 expression and VD3 treatment-induced increase in IGFBP-3 expression were optimized based on the protein concentration rather than the mRNA concentration. Meanwhile, Bcl-2 expression was down-regulated by VD3 treatment in an IGFBP-3-independent manner.

**Conclusion:**

These findings indicate the molecular mechanisms of IGFBP-3 induction stimulated by VD3 and IGFBP-3 independent Bcl-2 suppression by VD3 treatment in prostate cancer cells. The results could prompt a re-evaluation of VD3 usage in therapy for patients with prostate cancer.

## Background

Vitamin D has a central role in calcium and skeletal homeostasis [1, 2]. Its pleiotropic role both in physiological and pathological phenomena such as cell growth, immune function, and tumorigenesis has also been examined [3–6], which revealed that exposure of cancer cells to vitamin D significantly reduces the cell growth rate in multiple cancer types [6–8]. Indeed, recent epidemiological investigations have reported that higher vitamin D concentration could prevent multiple types of tumorigenesis [9]. Consistent with such finding, for example, an increase in colon cancer incidence with lower vitamin D dietary habits has been reported [10, 11]. However, suppressive effect on prostate cancer is still under discussion [12, 13].

Currently, prostate cancer is one of the most common cancers in men worldwide [14]. Clinical use of the prostate-specific antigen (PSA) test dramatically improved the screening sensitivity of prostate cancer compared to that of traditional methods; in turn, the number of patients with early-stage prostate cancer has rapidly increased since the mid-1990s [15, 16]. Unlike other cancer types, most cases of prostate cancer have slow progression or have non-progressive indolent symptom and are localized in the prostate; thus, they are unlikely to cause poor physical condition or death [16]. Therefore, patients with prostate cancer need a less burdensome treatment in order to avoid potential harm from excessive treatment.

Although the impact of vitamin D as a single agent on prostate cancer has been investigated, its significance remains under discussion [12, 13]. Meanwhile, the synergistic or additive effects of vitamin D and its derivatives, with anticancer drugs, on prostate cancer have been clinically studied, and encouraging results have been reported [17, 18]. However, the results of larger trials that evaluated the synergistic effect of vitamin D in combination with docetaxel, one of the first-line anticancer drugs in prostate cancer chemotherapy, showed limited or nonsignificant benefit of vitamin D efficacy in castration- or androgen deprivation therapy–resistant prostate cancer [19, 20]. Furthermore, overconsumption of 1α,25-dihydroxy vitamin D3 (VD3), also known as calcitriol, the biologically active form of vitamin D3, from food or prolonged treatment with VD3 derivatives could trigger hypercalcemia, resulting in physiological side effects [21]. Therefore, to date, VD3 has not been proactively used in the treatment of patients with prostate cancer. The biological function of vitamin D is mainly mediated by vitamin D receptor (VDR), which acts as a transcriptional factor [22]. Vitamin D receptor elements (VDRE) on the promoter region of target genes are recognized and transcriptionally activated by vitamin D–coupled VDR. Consistent with the diverse physiological function of VD3, VDRE was identified not only in the gene related to calcium and skeletal homeostasis but also in the gene related to fundamental cellular functions including cell growth [22]. IGFBP-3 is one of the families of six high affinity IGFBPs and was originally found in plasma as a stabilizer and transporter of IGFs in the bloodstream [23]. Interestingly, VDRE was found on the promoter of the *Igfbp-3* gene, and recent studies have revealed that IGFBP-3 functions inside the cell as well, regulating cell growth and apoptosis [24, 25].

## Methods

This study aimed to investigate IGFBP-3 induction by vitamin D treatment and determine its role in prostate cancer treatment with vitamin D in combination with anticancer drugs in order to provide molecular biological evidence of benefit of vitamin D and to suggest effective vitamin D usage in prostate cancer treatment.

### Chemicals and reagents

Dihydrotestosterone (DHT) and Calcitriol (VD3), purchased from Tokyo Chemical Industry (Tokyo, Japan), were resolved in ethanol as a stock solution. PEI MAX (molecular weight, 40,000) was purchased from Polysciences (PA, USA). The other chemicals and reagents were purchased from Wako Pure Chemical (Osaka, Japan) and Sigma-Aldrich (St Louis, MO, USA).

### Charcoal stripping of fetal bovine serum (FBS)

FBS was purchased from Gibco (Waltham, MA, USA). To deplete hormones, including testosterone, in FBS, dextran-coated charcoal powder was added to the serum, and the mixture was incubated with rotation at 4 degree overnight. Thereafter, the mixture was centrifuged to pellet charcoal, and the supernatant was filtered through a 0.22-μm polyvinylidene difluoride membrane. The charcoal-stripped serum was used for all experiments.

The concentrations of total testosterone and total vitamin D in the serum were determined using a total testosterone test kit (Abbott Japan, Chiba, Japan) and a total vitamin D test kit (Roche, Basel, Switzerland) according to manufacturers’ instructions. The concentrations of total testosterone in the pre- and post-treatment serum were 0.24 nM and less than 0.01 nM (limit of detection), respectively. The concentrations of total vitamin D in the pre- and post-treatment serum were 82.9 and 80.6 nM, respectively. Thus, the basal concentrations in the culture medium supplemented with 10% FBS were less than 0.001 nM total testosterone and approximately 8 nM total vitamin D.

### Cell culture

The LNCaP cell line was obtained from American Type Culture Collection and cultured in Dulbecco’s modified Eagle medium (DMEM; Sigma-Aldrich) supplemented with 10%charcoal-stripping FBS. The 293FT cell line was purchased from Invitrogen (Waltham, MA, USA) and cultured in DMEM supplemented with 10% FBS, 2 mM of l-glutamine, sodium pyruvate, and nonessential amino acids. The cells were cultured at a temperature of 37 °C in 5% CO_2_–humidified condition. The mycoplasma contamination was routinely checked and confirmed as negative.

### Cell growth assay

DMEM supplemented with charcoal-stripping FBS (10%) was used for the cell growth assay. The cells were seeded at 1 × 10^5^ cells per well in a 6-well plate. The next day, the medium was replaced with 2 mL of fresh medium, and 1 nM DHT and/or 10 nM VD3 were added. The cell culture was continued throughout the indicated period. The cultured cells were trypsinized, and the number of cells was assessed using an automated cell counter (Countess IITM FL; Invitrogen). Each assay was repeated at least three times, and similar results were obtained.

### Western blotting

The antibodies used for western blotting were mouse anti-IGFBP-3 (1:500; Santa Cruz Biotechnology), mouse anti-β-actin (1:1000; Santa Cruz Biotechnology), mouse anti-Bcl-2 (1:500; SantaCruz Biotechnology), and horseradish peroxidase–conjugated secondary antibodies (1:2500; Jackson ImmunoResearch Laboratories, West Grove, PA, USA). The collected cells were resuspended in RIPA buffer supplemented with protease and phosphatase inhibitors (Roche, Basel, Switzerland) and lysed using BIORUPTORTM II sonicator (Cosmo Bio, Tokyo, Japan). Cell lysates were resolved by 4–12% NuPAGE gels (Invitrogen) and transferred onto polyvinylidene fluoride membrane (Millipore, Burlington, MA, USA). The signals were developed using enhanced chemiluminescence reagent (PerkinElmer, Waltham, MA, USA), and LuminoGraph I (ATTO, Tokyo, Japan) was used for image capture. Quantification of band signal was analyzed using CS Analyzer 4 software (ATTO). At least two biological replicates of each experiment were performed, with similar results.

### Real-time reverse transcription–polymerase chain reaction

The total RNA from the cultured cells was extracted using TRIzol reagent (Invitrogen) according to the manufacturer’s instruction. The RNA was reverse transcribed by the PrimeScript RT reagent Kit (TaKaRa Bio, Shiga, Japan) using oligo-dT. Quantitative reverse transcription–polymerase chain reaction RT-PCR reaction was performed using TB Green Premix Ex Taq II (TaKaRa) and gene-specific primers (Supplementary Table 1) by CFX96 Touch Real-time PCR system (Bio-Rad Laboratories, Hercules, CA, USA). Gene expression data were normalized against glyceraldehyde 3-phosphate dehydrogenase or HPRT-1 as internal control. At least three biological replicates of each experiment were performed, and similar results were obtained.

### Flow cytometric analysis of cell cycle and apoptosis

For cell cycle analysis and apoptotic cell detection, flow cytometric analysis was performed using the Guava EasyCyte Plus flow cytometry system (Millipore) and Guava cell cycle reagent and Annexin V FITC apoptosis kit (Millipore) according to manufacturer’s instruction, as previously described [26]. At least three biological replicates of each experiment were performed, and similar results were obtained.

### Lentiviral construction and transduction

Backbone vectors, pLKO.1 puro plasmid (#8453), and pLenti CMV Puro DEST (w118–1) plasmid (#17452) were provided by Drs Bob Weinberg, Eric Campeau, and Paul Kaufman, respectively, via Addgene (Ref [27]). pENTR1A plasmid and lentiviral packaging mix (pLP1, pLP2, and pLP/VSVG) were purchased from Invitrogen. Full-length *Igfbp-3* was cloned from LNCaP cell complementary DNA (cDNA) using KOD FX neo (TOYOBO, Osaka, Japan) with a specific primer set (Supplementary Table 1) and inserted into the pENTR1A vector; then, the sequence was confirmed. The *Igfbp-3* cDNA was introduced into the pLenti CMV Puro DEST vector by recombinase reaction using LR Clonase II enzyme (Invitrogen) to generate the lentiviral expression vector. Specific short hairpin RNAs (shRNAs) were designed using Invitrogen or Biosettia websites. The selected target sequence oligos (Supplementary Table 1) were annealed and inserted into the pLKO.1 puro vector according to Addgene’s instruction. The lentiviral expression vector or shRNA vector was co-transfected with the lentiviral packaging mix into the 293FT cells using PEI MAX instead of Lipofectamine 2000 according to Invitrogen’s instruction. Twenty-four hours post-transfection, the medium was replaced with the culture medium for LNCaP cells. One day later, the lentivirus-containing supernatants were collected and filtrated through a 0.45-mm polyvinylidene fluoride filter (Millipore).

### Lentivirus infection

One day before infection, 1 × 10^5^ LNCaP cells were plated into a 10-cm dish; then, the culture medium was replaced with the lentivirus-containing supernatant, and culture was continued. Twenty-four hours post infection, the medium was replaced with fresh culture medium. Two days later, the medium was replaced with fresh culture medium that contained 1 μg/mL of puromycin, and culture was continued until the non-infected control cells were completely killed. The puromycin-selected cells were then subjected to each assay.

### Statistical analysis

Results are presented as mean ± standard deviation, unless otherwise specified. Pairs of groups were compared using a two-tailed unpaired Student’s t-test. One-way analysis of variance was used for multiple-group comparisons rather than specifying three. A *p*-value < 0.05 was considered statistically significant. All statistical analyses were performed using Excel software (Microsoft, Redmond, WA, USA) and Statcel3 add-in for Excel (OMS Publishing, Tokyo, Japan).

## Results

### VD3 reduces cell growth rate

**Fig. 1:**
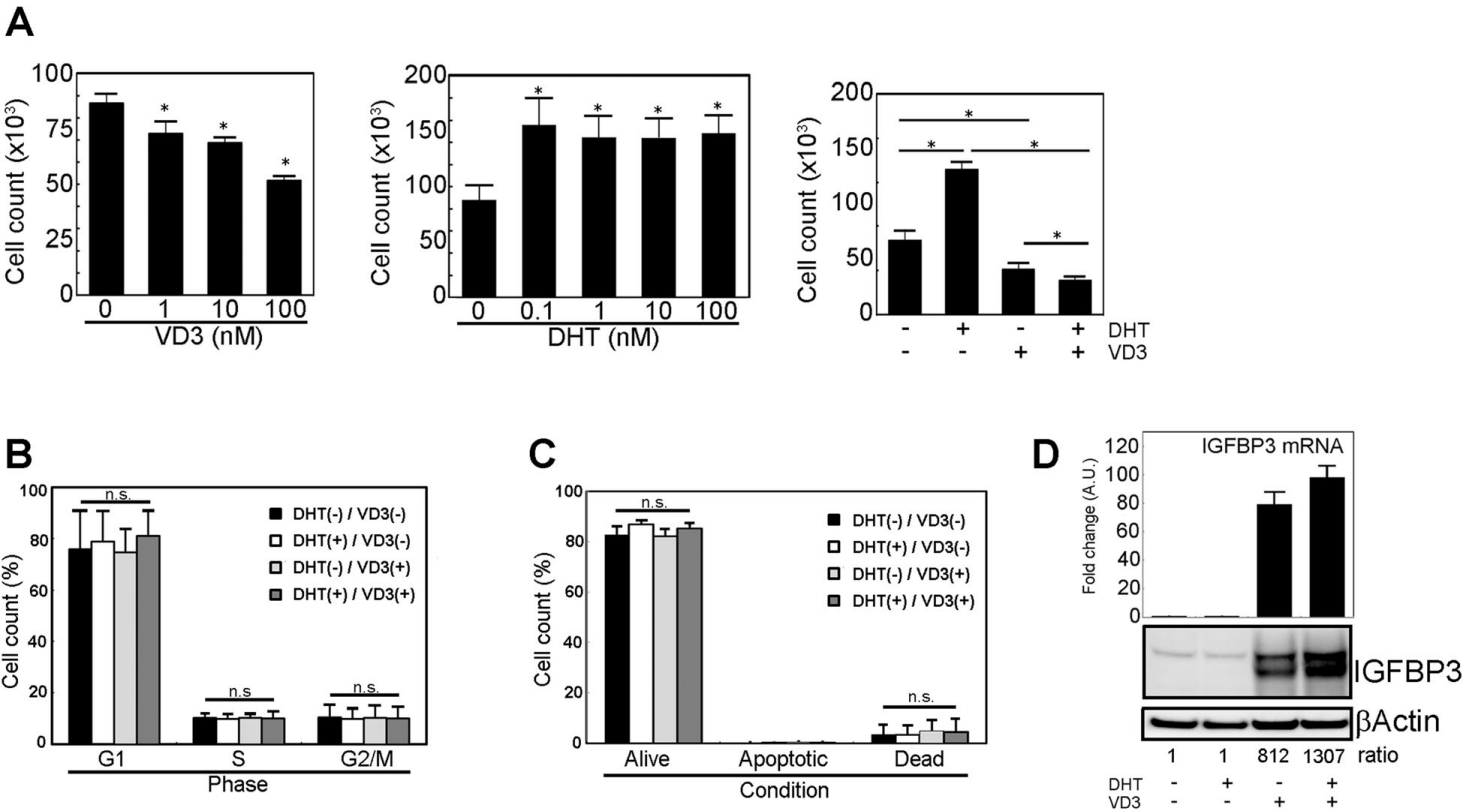
Cellular response of LNCaP cells treated with VD3 and DHT. **a** Effect of combined treatment of VD3 and DHT on cell growth in LNCaP cells: (left) VD3 treatment reduced cell growth in a dose-dependent manner; (center) DHT treatment increased cell growth at lower concentrations; (right) simultaneous treatment of VD3 and DHT enhanced the reduction of cell growth compared to treatment with VD3 alone. **b** Change in cell cycle phase by VD3 or DHT treatment. Neither VD3 nor DHT treatment significantly changed the cell cycle phase. **c** Induction of apoptosis by VD3 or DHT treatment. Neither low-dose VD3 nor DHT treatment influenced apoptosis at short-term. **d** Induction of IGFBP-3 expression by VD3 or DHT treatment. VD3 treatment-induced IGFBP-3 expression and co-treatment with DHT enhanced the expression level of IGFBP-3.The ratio indicates the density of IGFBP-3 band normalized by corresponding βActin band. The all experiments were performed in serum-containing medium condition. The uncropped full-length blot images are presented in Supplementary Fig. 5A

Consistent with a previous report [28] that treatment with VD3 inhibits growth of prostate cancer cells, our results showed that VD3 treatment reduced the cell growth rate in a dose-dependent manner (0, 1, 10 and 100 nM) (Fig. 1a, left). As shown below, IGFBP-3 induction activity of VD3 was reached to plateau at 5 nM concentration (Fig. 4a). On the other hand, testosterone has been reported to stimulate the growth of prostate cancer, and the results of this study confirmed that DHT treatment, from very low concentration (0.1 nM), stimulated cell growth rate and its activity was reached to plateau at less than 1 nM concentration (Fig. 1a, center). The purpose of our study was to investigate the role of VD3-IGFBP-3 induction system in cell growth inhibition and to propose the potency of low-dose VD3 usage which could evade side-effect of VD3 treatment such as hypercalcemia in therapy for patients with prostate cancer, thus 1 nM of DHT and 10 nM of VD3 concentrations were chosen as minimum but stably working concentration for following experiment. Previously, it has been demonstrated that simultaneous treatment with VD3 and DHT enhanced the reduction of cell growth rate compared to treatment with VD3 alone, and a similar result was reproduced with low-dose DHT (1 nM) and VD3 (10 nM) in this study (Fig. 1a, right). To further characterize growth inhibitory effect with low-dose of DHT (1 nM) and VD3 (10 nM), cell cycle and apoptotic analyses were performed with flow cytometry. The cell cycle analysis revealed that there was no significant change in the cell cycle phase distribution among control (no treatment), DHT (1 nM), VD3 (10 nM), and DHT/VD3 treatment conditions (Fig. 1b), suggesting that low-dose of VD3 or DHT/VD3 treatment did not arrest the cell cycle at a specific phase. Previous reports have shown that long-term VD3 treatment has apoptosis inducible activity [29], and DHT has apoptosis inhibiting activity in a dose-dependent manner [30] in LNCaP cells. However, it was unclear or controversial whether lower-concentration of VD3 and DHT at short-term could influence apoptosis, respectively, in LNCaP cells. Thus, the apoptosis assay was performed with 1 nM of DHT and 10 nM of VD3 for short-term, and found that neither lower-dose DHT, VD3 nor DHT/VD3 treatment for short-term influenced apoptosis (Fig. 1c). These results suggested that the decrease in the cell number induced by low-dose VD3 or DHT/VD3 treatment was mainly due to a decrease in the cell growth rate. To further address what was occurring at the molecular level during low-dose DHT and/or VD3 treatment, the genes known to regulate the cell cycle and inducible by DHT and/or VD3 treatment were chosen, and messenger RNA (mRNA) induction was quantitatively measured (Fig. 1d upper, Supplementary Fig. 1A). The quantitative RT-PCR results showed that Igfbp-3 mRNA induction was positively correlated to cell growth suppression in response to low-dose VD3 or DHT/VD3 treatment, and the expression strength was dramatically sensitive to VD3 or DHT/VD3 treatment. Consistent with that, IGFBP-3 protein was markedly induced by VD3 treatment and it was enhanced by simultaneous treatment of VD3 with DHT. A similar response was observed in the expression of AR, the receptor of DHT, which was known to a one of target of VDR (Supplementary Fig. 1B).

Multiple recent studies have revealed that IGFBP-3 functions in cellular response, including cell growth and apoptosis, in an insulin-like growth factor (IGF)-independent manner. Considering these findings, we believe that IGFBP-3 can be a key molecule for VD3 treatment in prostate cancer cells.

### IGFBP-3 was a dominant factor in cell growth suppression

**Fig. 2:**
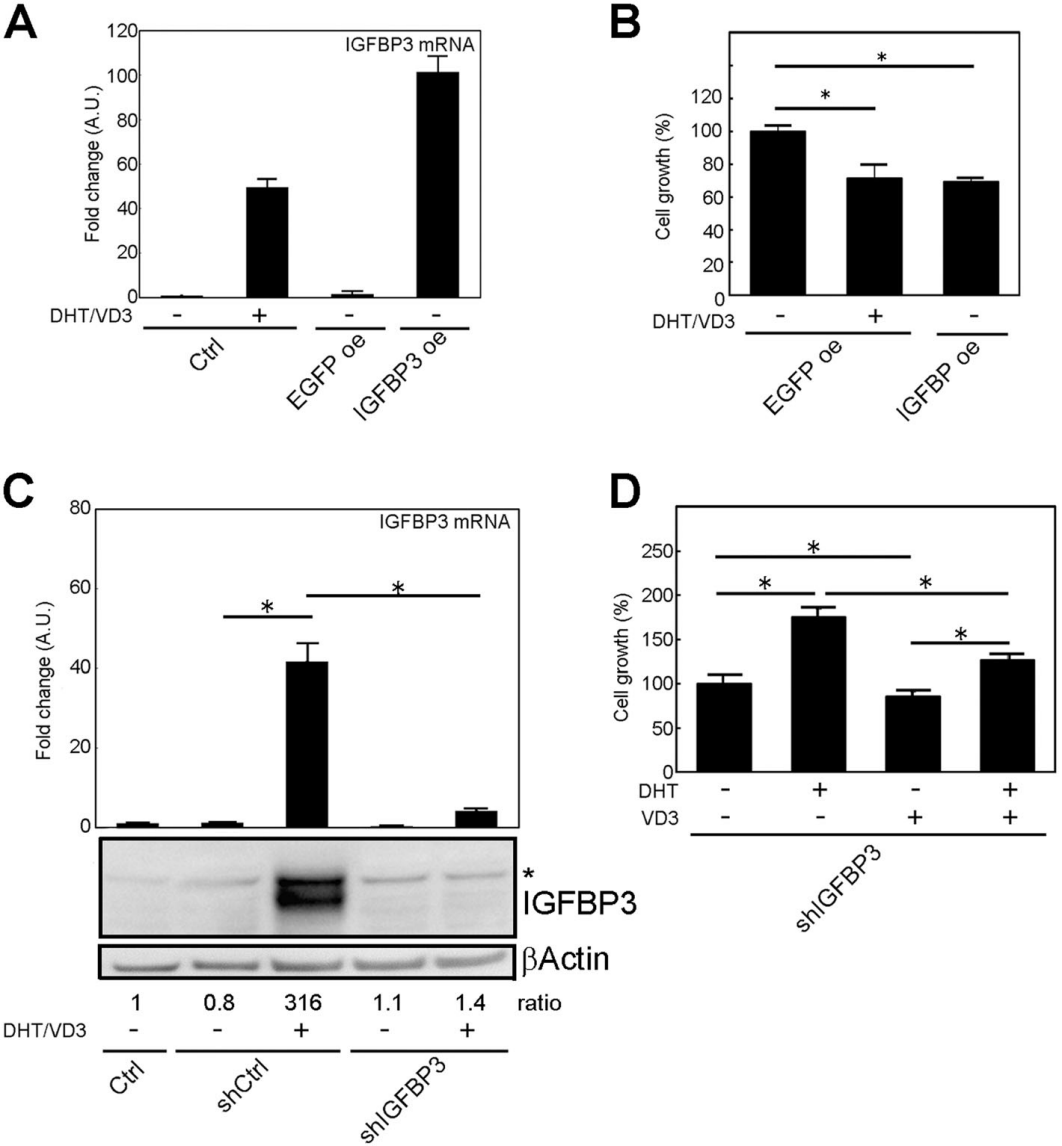
IGFBP-3 mediates the effect of VD3 on cell growth in LNCaP cells. **a** Overexpression of IGFBP-3 in LNCaP cells. LNCaP cells were infected with lentivirus containing the *IGFBP-3* gene, and its overexpression was confirmed by quantitative reverse transcription–polymerase chain reaction. **b** Suppression of cell growth by IGFBP-3. IGFBP-3–overexpressed cells were cultured as they were and control cells were cultured with DHT/VD3 for 3 days, and then the cell number was measured. **c** Knockdown of IGFBP-3 in LNCaP cells. LNCaP cells were infected with lentivirus containing shRNA for Igfbp-3, and IGFBP-3 knockdown was confirmed by quantitative reverse transcription–polymerase chain reaction and western blotting. The ratio indicates the density of IGFBP-3 band normalized by corresponding βActin band. **d** The IGFBP-3 knockdown cells were treated with DHT and/or VD3 for 3 days, and then the cell number was measured. The all experiments were performed in serum-containing medium condition. The uncropped full-length blot images are presented in Supplementary Fig. 5B

To confirm if IGFBP-3 dominantly suppresses cell growth in LNCaP cells, we applied the gain-of-function and loss-of-function approach using a lentivirus system. First, we generated IGFBP-3–overexpressing LNCaP cells and found that the expression of IGFBP-3 mRNA was about 50% higher compared to that by low-dose DHT/VD3 treatment (Fig. 2a). As an infection control, EGFP-overexpressing LNCaP cells were also generated, and it was confirmed that lentivirus infection per se did not induce IGFBP-3 expression. Using these cell lines, the effect of IGFBP-3 on cell growth was observed (Fig. 2b). Results showed that the cell number of EGFP-overexpressed cells treated with 1 nM of DHT and 10 nM of VD3 for 3 days was decreased to 70% compared with that of untreated cells, and the IGFBP-3–overexpressing cells showed comparable cell growth decrease without DHT/VD3 treatment. Next, we generated shRNA for Igfbp-3 (shIgfbp-3)–expressing LNCaP cells. The knockdown of IGFBP-3 mRNA and protein induced by low-dose DHT/VD3 treatment was confirmed in shIgfbp-3–expressing LNCaP cells (Fig. 2c). Using this cell line, the effect of low-dose DHT and VD3 treatment on cell growth was observed. As we expected, the suppressive efficacy of low-dose VD3 on cell growth was weakening, and simultaneous treatment with 1 nM of DHT and 10 nM of VD3 increased cell growth (Fig. 2d). Taken together, these data indicated IGFBP-3–dominant factor of cell growth suppression induced by low-dose VD3 treatment in LNCaP cells.

### Acceleration of anticancer drug effect by VD3

**Fig. 3:**
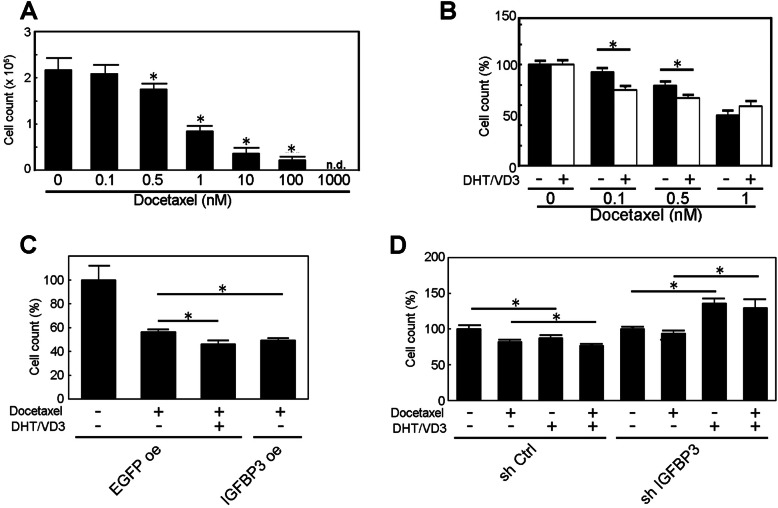
VD3 enhanced cytotoxicity of docetaxel. **a** Dose-dependent cytotoxicity of docetaxel. LNCaP cells were treated with docetaxel for 3 days, and then the living cell number was measured. IC50 was 0.82 nM. **b** Simultaneous treatment of VD3 with dose-dependent docetaxel. Simultaneous treatment of VD3 with docetaxel reduced the living cell number at 0.1–0.5 nM range. **c** IGFBP-3 overexpression was conducive to DHT/VD3 treatment on docetaxel treatment. IGFBP-3–overexpressed or control cells were treated with DHT/VD3 and 0.1 nM of docetaxel and cultured for 3 days, and then, the cell number was measured. **d** IGFBP-3 knockdown canceled the effect of VD3 on docetaxel treatment. In IGFBP-3 knockdown cells, DHT/VD3 treatment increased the cell number compared to that of the no-treatment control, and the cell number was almost equal to that of the DHT treated sample. The concentration of docetaxel used was 0.1 nM. The all experiments were performed in serum-containing medium condition

As previously reported and we demonstrated above, VD3 alone is not cytotoxic at physiological and pharmacological concentrations. Meanwhile, simultaneous treatment with VD3 has been reported to improve the efficacy of anticancer drugs including docetaxel, however its molecular mechanisms were remained not fully uncovered. Here, we supposed that IGFBP-3 might be a mediator of VD3-induced sensitization to anticancer drugs in prostate cancer cells. To confirm this hypothesis, LNCaP cells were treated with low-dose of DHT/VD3 in combination with several concentrations of docetaxel. To determine the appropriate concentration of docetaxel for evaluating the VD3 effect, we first screened the concentration of docetaxel based on cytotoxic activity and found that a docetaxel concentration > 10 nM killed bulk of the cells treated (Fig. 3a). Here, IC50 of docetaxel was 0.82 nM in our assay, and it was consistent with previous reports (0.44–1.6 nM). Thus, a docetaxel concentration < 0.1 nM was chosen to observe the effect of combinatorial low-dose DHT/VD3 treatment for the following assay. To evaluate the synergistic effect of DHT/VD3 on cytotoxicity by docetaxel, LNCaP cells were treated with 0, 0.1, 0.5, or 1 nM of docetaxel with or without DHT/VD3, and results showed that low-dose DHT/VD3 with docetaxel reduced the living cell number at the concentration range of 0.1–0.5 nM, but the effect was masked when 1 nM docetaxel was applied (Fig. 3b). Similarly, low-dose DHT/VD3 with Cisplatin reduced the living cell number at the concentration range of 1–10 nM (Supplementary Fig. 2). To see if these enhanced cytotoxicity effects were dependent on IGFBP-3, IGFBP-3–overexpressed or shIgfbp-3–expressed LNCaP cells were analyzed in the same manner. Indeed, in IGFBP-3–overexpressed cells, the living cell number was reduced by docetaxel without DHT/VD3 addition (Fig. 3c). Correspondingly, in the shIgfbp-3-expressed cells, reduction of living cell number by DHT/VD3 addition was canceled; rather, the cell living number was increased (Fig. 3d). The increase of living cell number despite DHT/VD3 addition in docetaxel treated shIgfbp-3-expressed cells was assumed by cancelation of VD3 effect and emerging of DHT effect on cell growth. Based these findings, low-dose DHT/VD3–induced enhanced cytotoxicity by docetaxel on LNCaP cells was dependent on IGFBP-3 expression.

### Characterization of the IGFBP-3 induction mechanism

**Fig. 4:**
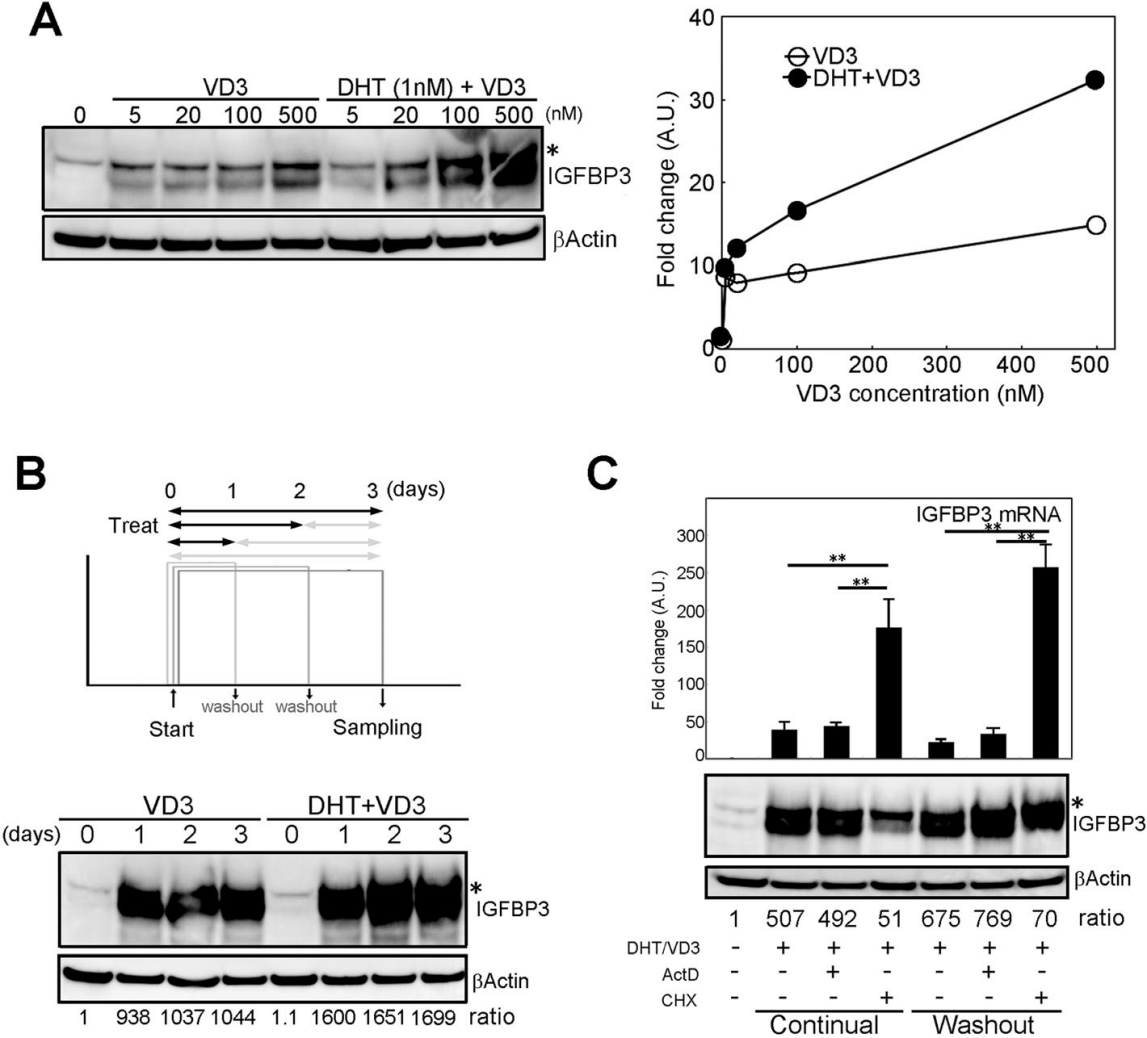
Characterization of IGFBP-3 expression induced by VD3 and DHT treatments. **a** Effect of dose-dependent VD3 treatment with or without DHT on IGFBP-3 induction. LNCaP cells were treated with VD3 alone at the indicated concentration or with VD3 and 1 nM DHT: (top) western blotting image of IGFBP-3 and (bottom) quantified graph of IGFBP-3 induction. Open circles; VD3 alone, filled circles; DHT + VD3. **b** Effect of temporal treatment of DHT/VD3 on IGFBP-3 induction: (top) schematic time course of temporal treatment of VD3 and (bottom) western blotting image of IGFBP-3. The ratio indicates the density of IGFBP-3 band normalized by corresponding βActin band. **c** Effect of mRNA transcription and protein synthesis on the stability of IGFBP-3 expression induced by DHT/VD3. LNCaP cells were treated with VD3/DHT for 1 day, followed by actinomycin D (ActD; 5 μM) or cycloheximide (CHX; 10 μM) for another day after VD3/DHT was washed out or left as is: (top) quantification graph of Igfbp-3 mRNA induction, (middle) western blotting image of IGFBP-3 and the combination of treatments (bottom). The ratio indicates the density of IGFBP-3 band normalized by corresponding βActin band. The all experiments were performed in serum-containing medium condition. The uncropped full-length blot images are presented in Supplementary Fig. 5C

As demonstrated above, IGFBP-3 had a pivotal role in low-dose DHT/VD3-induced enhanced cytotoxicity by antitumor drugs. To further dissect the IGFBP-3 induction mechanism and to provide the molecular evidence of VD3 treatment for clinical research, mechanisms of IGFBP-3 induction by DHT and VD3 were analyzed. As previously reported in prostate cancer cells, VD3 treatment induces CYP24A1 as well, an enzyme that catalyzes VD3 to its inactive form. As a negative feedback factor, the induced CYP24A1 limits the efficacy of VD3. Meanwhile, activated AR induced by DHT treatment suppresses CYP24A1 transcription, thus cancelling the negative-feedback loop to inactivate VD3. Consistent with that, CYP24A1 induction by VD3 treatment and its suppression by simultaneous treatment with DHT were confirmed even when we applied low-dose DHT and VD3, which were enough to induce and suppress CYP24A1 expression (Supplementary Fig. 3), meaning that h low-dose DHT/VD3 treatment could cancel the CYP24A1-driven negative-feedback loop. To further dissect the mechanism of IGFBP-3 expression in LNCaP cells, the cells were treated with VD3 alone or DHT (fixed in 1 nM) and VD3 in a dose-dependent manner (0, 5, 20, 100, and 500 nM). When treated with VD3 alone, the induced IGFBP-3 reached a plateau at 5 to 20 nM. In contrast, when treated with VD3 together with DHT, the amount of induced IGFBP-3 was increased according to the increment of VD3 concentration (Fig. 4a). These results indicated that low-dose DHT could improve IGFBP-3 induction activity of VD3 through CYP24A1 suppression.

Clinically, high-dose VD3 or its derivatives for treatment can cause hypercalcemia; thus, its continual usage should be carefully monitored to avoid side-effect of VD3. Here, we wondered if continual VD3 treatment would be required for maintaining IGFBP-3 induction in prostate cancer cells. To address this, LNCaP cells were treated with VD3 alone or low-dose DHT/VD3 for 1, 2, or 3 days, followed by washout, which was done by replacing the culture medium and continuing the culture for 3 days in total (Fig. 4b, top). Here, intracellular IGFBP-3 protein was observed by western blotting. Interestingly, 1-day treatment of VD3 or DHT/VD3 induced stable IGFBP-3 expression (Fig. 4b, bottom), although treatment of VD3 alone showed mild IGFBP-3 induction compared to that by DHT/VD3. Note that, IGFBP-3 showed similar strength of expression between 1 and 3 days of VD3 or DHT/VD3 treatment. This result clearly indicated that temporal VD3 treatment could induce prolonged stable IGFBP-3 expression.

This nonlinear response suggested the presence of a unique molecular property underlying the IGFBP-3 expression mechanism. Generally, nonlinear cellular response such as sustained protein expression by temporal stimulation was triggered by positive-feedback loop mechanism, in which protein synthesis and transcriptional regulation was included as hysterical response driver. Thus, to further dissect if protein synthesis or transcriptional regulation, or both are involved in nonlinear VD3-IGFBP-3 induction, actinomycin D and cycloheximide, which are transcriptional inhibitor and protein synthesis inhibitor, respectively, were added with VD3 or DHT/VD3, and behavior of IGFBP-3 protein and its mRNA was observed. Experimentally, actinomycin D or cycloheximide was added 1 day after treatment with VD3 alone or DHT/VD3, and the culture was continued for another day. When interfered with 5 μM of actinomycin D, there was no change in IGFBP-3 mRNA or protein expression (Fig. 4c). By contrast, when interfered with 10 μM of cycloheximide, the IGFBP-3 protein was immediately reduced, however, the mRNA was unexpectedly increased several times higher than those without cycloheximide interference (Fig. 4c). The unexpected mRNA increase became stronger when DHT/VD3 washout was performed ahead of the cycloheximide interference. These cellular responses on IGFBP-3 induction interfered by Actinomycin D and cycloheximide suggested that the cells had a protein abundance–based positive-feedback loop to maintain the total amount of IGFBP-3 via transcriptional control.

### IGFBP-3–independent Bcl-2 suppression by VD3

**Fig. 5:**
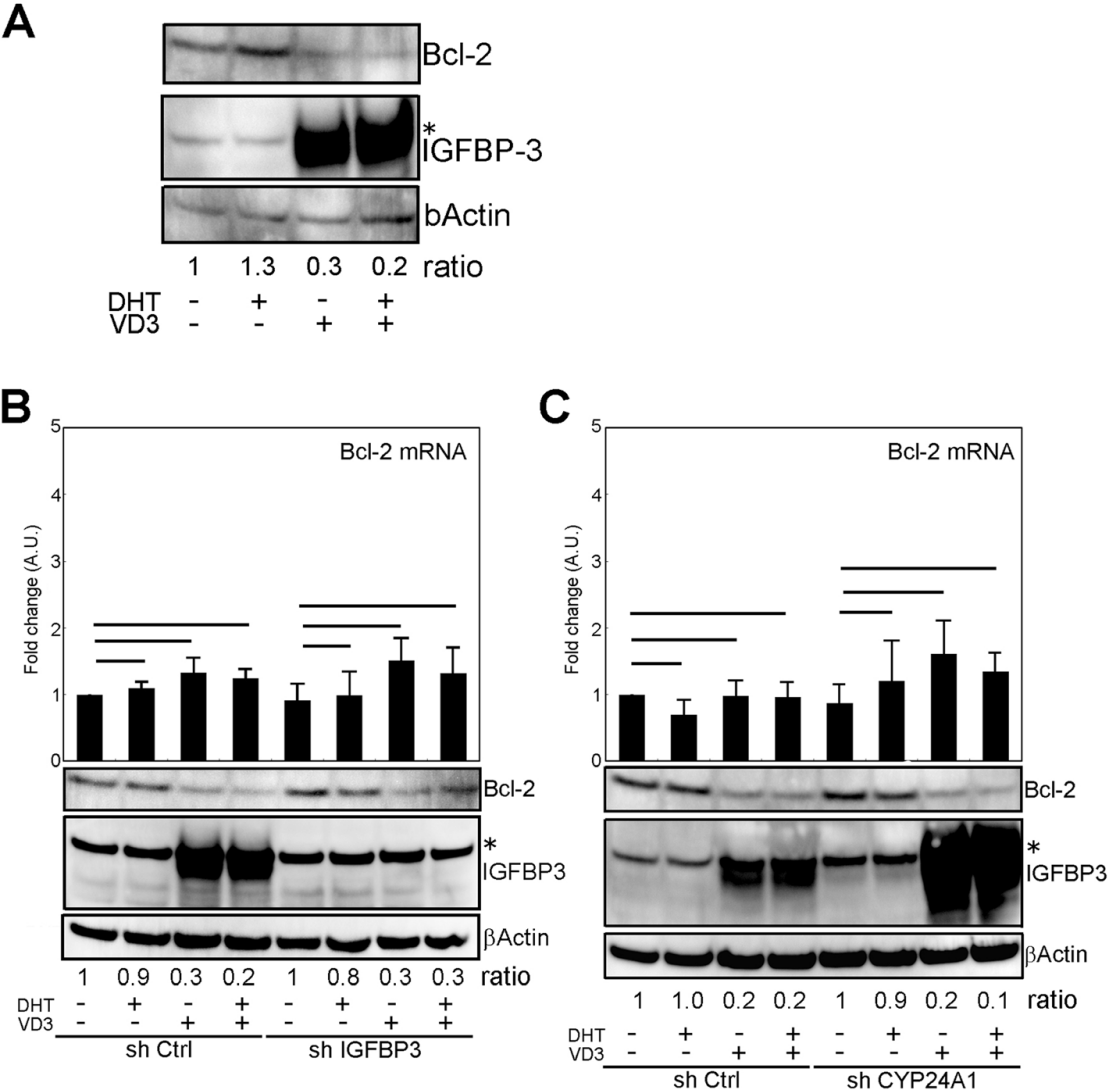
IGFBP-3–independent reduction of Bcl-2 protein expression induced by VD3. **a** Western blotting image of Bcl-2 reduction by VD3 treatment. The ratio indicates the density of Bcl-2 band normalized by corresponding βActin band. **b** Effect of IGFBP-3 suppression on Bcl-2 reduction by VD3 treatment. The cells were infected with lentivirus encoding of shRNA for Igfbp-3, and then treated with VD3 and DHT: (top) quantification graph of Bcl-2 mRNA expression and (bottom) western blotting image of Bcl-2 and IGFBP-3 induced by VD3 and DHT treatment. The ratio indicates the density of Bcl-2 band normalized by corresponding βActin band. **c** Effect of IGFBP-3 overexpression on Bcl-2 reduction by VD3 treatment. The cells were infected with lentivirus encoding of the *Cyp24a1* gene; then, the cells were treated with VD3 and DHT: (top) quantification graph of Bcl-2 mRNA expression and (bottom) western blotting image of Bcl-2 and IGFBP-3 induced by VD3 and DHT treatment. The ratio indicates the density of Bcl-2 band normalized by corresponding βActin band. The all experiments were performed in serum-containing medium condition. The uncropped full-length blot images are presented in Supplementary Fig. 5D, E

As shown above, although low-dose DHT and/or VD3 treatment did not induce apoptosis (Fig. 1d), VD3 treatment rendered LNCaP cells sensitive to the antitumor drugs (Fig. 3b and Supplementary Fig. 2), suggesting that any apoptosis-related factor might be influenced. To address this idea, we investigated the behavior of apoptosis-related molecules in response to low-dose VD3/DHT treatment. Consistent with previous report [29], although the concentration of VD3 was higher than that we used here, Bcl-2 protein, an anti-apoptotic molecule, was down-regulated by low-dose VD3 treatment (Fig. 5a). Compared to that by VD3 alone, it seemed that Bcl-2 down-regulation by DHT/VD3 was not seemed to be enhanced unlike to IGFBP-3 expression, suggesting that Bcl-2 down-regulation by VD3 was IGFBP-3 induction independent manner. To see whether Bcl-2 down-regulation was IGFBP-3–dependent or not, the behavior of the expression of Bcl-2 protein and mRNA was observed in shIGFBP-3expressedcells. Interestingly, despite the IGFBP-3 disappearance, Bcl-2 down-regulation was observed according to VD3 or DHT/VD3 treatment (Fig. 5b, bottom), and it was not significantly different at protein and mRNA expression level compared to that in shCtrl-expressing cells. Moreover, in order to confirm that in IGFBP-3 expression-enhanced condition, shCYP24A1 expression cells were established in which VD3 effect on IGFBP-3 induction was expected to strengthen. The knockdown of Cyp24a1 under the VD3 treatment condition was confirmed by qRT-PCR (Supplementary Fig. 4). Indeed, the amount of IGFBP-3 protein was increased in shCYP24A1-expressing cells compared to that in shCtrl cells (Fig. 5c, bottom). Despite enhancement of IGFBP-3 expression, down-regulation Bcl-2 protein by low-dose VD3 or DHT/VD3 treatment was not observed compared to that in shCtrl cells (Fig. 5c, bottom). Also, the expression of Bcl-2 mRNA was not significantly changed (Fig. 5c, top). These results suggested that the down-regulation of Bcl-2 protein by low-dose VD3 treatment was independent of IGFBP-3 induction. Besides, the mRNA of Bcl-2 was not significantly changed according to VD3 or DHT/VD3 treatment even when IGFBP-3 expression was modified (Fig. 5b, top and c, top), suggesting that this event was unlikely to depend on transcriptional regulation but rely on protein synthesis and/or degradation mechanism. Taken together, it was thought that low-dose VD3 treatment suppressed Bcl-2 expression at protein level in an IGFBP-3 induction independent manner.

### Model of VD3 function in prostate cancer treatment

**Fig. 6:**
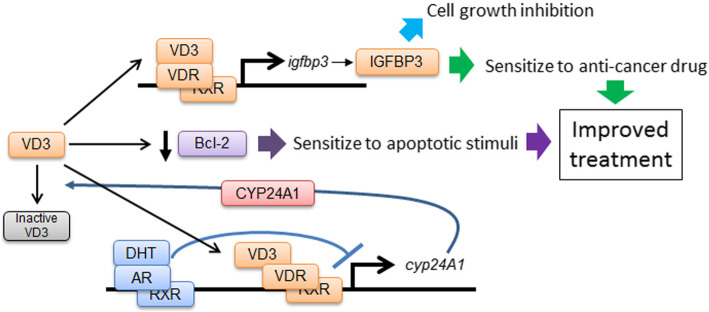
Schematic diagram of the molecular basis of VD3 treatment. VD3 treatment sensitizes the treatment of anticancer drugs in an IGFBP-3–dependent manner. DHT treatment enhances IGFBP-3 expression through the suppression of CYP24A1 induction. VD3 treatment also reduces Bcl-2 protein expression in an IGFBP-3–independent manner

Considering these findings, it was expected that low-dose VD3 treatment could provide an advantage in the treatment of prostate cancer cells through IGFBP-3–dependent and IGFBP-3–independent manner. In addition, we uncovered the unique property of IGFBP-3 induction by which temporal VD3 treatment could induce sustained prolonged IGFBP-3 expression, allowing reduction of the amount of VD3 usage, by that could prevent side-effect of VD3 treatment. These findings are summarized in Fig. 6.

## Discussion

In this study, we demonstrated in LNCaP cells that simultaneous treatment of low-dose VD3 could enhance the cytotoxicity of anticancer drugs at a lower concentration and that the effect of VD3 was via IGFBP-3 induction. Furthermore, the induction system for IGFBP-3 by VD3 had a unique property, in which temporal VD3 treatment could induce prolonged IGFBP-3 induction and it appeared to be able to sustain IGFBP-3 expression with positive feedback via transcriptional control. Besides, VD3 treatment can suppress Bcl-2 expression in an IGFBP-3–independent manner. Overall, our results provided the evidence of the molecular mechanisms of VD3 efficacy in the treatment of prostate cancer.

Several opponent studies regarding IGFBP-3 on growth inhibition in prostate cancer cells have been reported. Boyle et al. demonstrated growth inhibitory effect of IGFBP-3 via secretion to culture media (34). On the other hand, Stewart and Weigel have shown that IGFBP-3 induced by VD3 was not required for cell growth inhibition (35), and they mentioned culture condition with or without serum in which IGFs could cause different results. Here, our data stand for the conclusion of Boyle et al. even in serum-containing culture condition and inconsistent with the conclusion of Stewart and Weigel. In this study, we focused on efficacy of low-dose VD3 that would not induce apoptosis and low concentration of docetaxel that slightly could kill LNCaP cells, based on MIGFBP-3 expression as functional mediator on cell growth inhibition. In addition, the different VD3 concentration could cause different induction pattern of IGFBPs including IGFBP2 and − 3 under different culture conditions. Previously, it has been reported that higher concentration of synthetic androgen analogue (R1881) or DHT treatment also induced IGFBP-3 expression in prostate cancer cells [31, 32]. Also, enhanced cell growth by exogenous IGFBP-3 treatment has demonstrated [31], although it was opponent role of IGFBP-3 on cell growth in other reports [33, 34]. After all, regarding IGFBP-3 function on cell growth, growth inhibitory, growth stimulatory and non-effective role have been suggested. Among them, our results stand for the first one. Further study that dissects relationship among VD3 or DHT concentration, induction pattern of IGFBPs and their efficacy on the cell growth inhibition would be required in depth understanding of the VD3 and DHT treatment in combination with anticancer drugs and its molecular mechanisms on cell growth inhibition in prostate cancer cells. The key is how IGFBP-3 functions in patient’s tumor. Molecular dissect of IGFBP-3 function on cell growth in prostate cancer cells in vitro and monitoring of IGFBP-3 in response to VD3 treatment, castration or androgen-deprivation therapy in patient’s prostate tumor would be future challenge.

Although epidemiological studies have indicated the benefit of using VD3 in the preventing tumorigenesis in multiple cancer types, it benefit in prostate cancer prevention is unclear [9, 12–14]. Meanwhile, the efficacy of VD3 in prostate cancer chemotherapy has been expected, but its efficacy as a single agent has not been determined and its use as a concomitant drug with anticancer drugs remains under discussion [20]. In most cases, the concentration of anticancer drugs used in the trial was high enough to produce a significant effect even when used as a single agent; thus, whether the dose of the anticancer drugs used in the trial might be too high to evaluate the efficacy of VD3 is a concern. The essence of VD3 usage in combination with anticancer drugs is perhaps to reduce the concentration of anticancer drugs, thereby decreasing their adverse effects.

Previous clinical trials of VD3 on prostate cancer treatment had suggested limited VD3 efficacy in androgen-deprivation therapy– or castration-resistant prostate cancer treatment [19, 20]. Based on our results, the resistance could be explained in part by the limited IGFBP-3 induction by VD3 because of the failure to suppress CYP24A1 by DHT via AR. The trial study of CYP24A1 inhibitor in prostate cancer treatment has been examined to improve VD3 efficacy [35]. Measuring IGFBP-3 induction by VD3 would be a good index to evaluate the efficacy of the CYP24A1 inhibitor.

In this study, we uncovered the unique property of the IGFBP-3 induction mechanism, the nonlinear response of IGFBP-3 induction by DHT/VD3, and the feedback loop to sustain IGFBP-3 expression in LNCaP cells. Successive treatment with VD3 or its derivatives is known to cause hypercalcemia. By contrast, our data indicated that temporal VD3 treatment was enough to sustain prolonged IGFBP-3 expression, suggesting that a temporal higher concentration, rather than a constant concentration, of VD3 may be important in maximizing its efficacy in prostate cancer treatment. Here, we observed intracellular IGFBP-3 expression and its mRNA to dissect what mechanisms were contributed in sustained IGFBP-3 expression. Originally, IGFBP-3 was found as binding partner of IGFs in blood stream. Further analysis if the amount of secreted IGFBP-3 was in parallel with that of intracellular IGFBP-3 to dissect the spatio-temporal function of IGFBP-3 would be important for in depth understanding how IGFBP-3 contributes in cell growth inhibition in patients with prostate cancer. Our data can serve as basis to reduce the total amount of VD3 in the prostate cancer treatment. Although the physiological mechanisms are unclear, these characteristics would be beneficial in VD3 usage in prostate cancer treatment.

Generally, up-regulation of Bcl-2 expression is found in solid tumors including prostate cancer, and it is known to be one of the causes of resistance against radiation therapy [36]. Previously, Bcl-2 down-regulation by VD3 treatment at higher concentration has been reported [29]. Consistent with that, our results showed that even low-dose VD3 treatment decreased Bcl-2 protein expression in LNCaP cells. In addition, we found that it was an IGFBP-3–independent manner and transcriptional change of Bcl-2 mRNA did not occur, suggesting that Bcl-2 down-regulation by VD3 treatment was protein synthesis- or degradation-related issue. The suppressive effect of VD3 treatment on Bcl-2 protein may be standing on the non-canonical pathway of VD3-VDR axis. From another perspective, VD3 treatment can provide benefit on radiation therapy in androgen deprivation therapy– or castration–resistant prostate cancer identical to typical ones. Indeed, pathologically, Bcl-2 overexpression is typically observed in tumor-wide and it is well known to be a cause of treatment-resistant, meanwhile, Bcl-2 suppression by VD3 has been reported in multiple tumor types including prostate cancer [32, 37–39], suggesting that the relationship between VD3 and Bcl-2 could be common in tumor-wide. Although accumulated evidence suggests the existence of a molecular link between VD3 and Bcl-2 in multiple tumor types, proapoptotic effects of VD3 remain to be elucidated at the cell biological level and in animal studies for each tumor type, including prostate cancer. Further biological and preclinical studies aimed at promoting the proapoptotic activity of VD3 may help establish the significance of VD3 in cancer treatment.

In this study, we demonstrated all the experiments through one of the typical prostate cancer cell lines, LNCaP cells, thus additional study with cell line-wide and, if possible, patient-derived primary culture would be needed to perceive how universal our finding is in prostate cancer. Further study in-depth analysis would be a next challenge.

## Conclusion

Our data provided molecular insight on the use VD3 in the treatment in prostate cancer cells, encouraging further clinical study to evaluate VD3 efficacy in patients with various types of prostate cancer including radiation-, castration-, and androgen deprivation therapy–resistant ones.

## Supplementary information

**Additional file 1: Supplementary Table 1.** Sequence information of primers and oligos. Primer set used for real-time-RT-PCR and the oligo DNA sequence for shRNA expression vector construction are listed.

**Additional file 2: Supplementary Fig. 1.** Expression profiling of the genes induced by VD3 and/or DHT treatment. (a) Expression profiling of the genes related to cellular response that have VDR responsible element on their promoter in LNCaP cells. IGFBP-3 mRNA dramatically responded to VD3 treatment compared to the other genes tested. (b) Expression profiling of the genes related to the VD3 signaling cascade. Androgen receptor (AR), the receptor of DHT, was induced by VD3 treatment.

**Additional file 3: Supplementary Fig. 2.** Cisplatin dose-dependent cytotoxicity in LNCaP cells. LNCaP cells were treated with low-dose cisplatin at the indicated concentration with DHT/VD3. The cells were cultured for 3 days; then, the cell number was measured. At a concentration range of 1–10 nM, DHT/VD3 enhanced the cytotoxicity of cisplatin.

**Additional file 4: Supplementary Fig. 3.** Establishment of shCYP24A1 expressing LNCaP cells. LNCaP cells were infected with lentivirus that carried shCYP24A1, selected with puromycin, then subjected to VD3 treatment. mRNA of Cyp24a1 was quantitatively observed by qRT-PCR.

**Additional file 5: Supplementary Fig. 4.** Induction of the *CYP24A1* mRNA expression in LNCaP cells in response to low-dose DHT and VD3 treatment. The expression of *CYP24A1* mRNA was induced by low-dose VD3 (10 nM) and was suppressed by simultaneous treatment with DHT (1 nM).

**Additional file 6: Supplementary Fig. 5.** Original uncropped images of western blot.

## Data Availability

The materials constructed by the authors and data analyzed during the current study are available from the corresponding author on reasonable request.

## Abbreviations

VD3: 1α,25-dihydroxy vitamin D3
DHT: Dihydrotestosterone
IGFBP-3: Insulin-like growth factor binding protein-3
cDNA: Complementary DNA
DMEM: Dulbecco’s modified Eagle medium
FBS: Fetal bovine serum
shRNA: Short hairpin RNA
VDR: Vitamin D receptor
VDRE: Vitamin D receptor elements
