# Supplementary material for: Molecular evidence of IGFBP-3 dependent and independent VD3 action and its nonlinear response on IGFBP-3 induction in prostate cancer cells
Source: BMC Cancer. 2020 Aug 24;20:802. doi: 10.1186/s12885-020-07310-5 (PMC7446217; doi:10.1186/s12885-020-07310-5)
Supplement: Supplementary file 1 — Additional file 1: Supplementary Table 1. Sequence information of primers and oligos. Primer set used for real-time-RT-PCR and the oligo DNA sequence for shRNA expression vector construction are listed. [file 12885_2020_7310_MOESM1_ESM.docx]

Supplementary Table 1

| **Primer/ shRNA oligo** | **Sequence (5’-3’)** |
| --- | --- |
| *For real-time RT-PCR* | |
| Igfbp-3 forward | AGAGCACAGATACCCAGAACT |
| Igfbp-3 reverse | GGTGATTCAGTGTGTCTTCCATT |
| CYP24A1 forward | GGTGGCGAGACTCAGAACG |
| CYP24A1 reverse | GTCGTGCTGTTTCTTGAGACC |
| p27^Kip1^ forward | AACGTGCGAGTGTCTAACGG |
| p27^Kip1^ reverse | CCCTCTAGGGGTTTGTGATTCT |
| CDK2 forward | CCAGGAGTTACTTCTATGCCTGA |
| CDK2 reverse | TTCATCCAGGGGAGGTACAAC |
| HOXC8 forward | ACCGGCCTATTACGACTGC |
| HOXC8 reverse | TGCTGGTAGCCTGAGTTGGA |
| GNMT forward | CTGGGGTGGACTCCATTATGC |
| GNMT reverse | GATGACCCACTTGTCGAAGGC |
| IVL forward | TCCTCCAGTCAATACCCATCAG |
| IVL reverse | CAGCAGTCATGTGCTTTTCCT |
| PTH forward | ATTTACGGCGACGATTCTTCC |
| PTH reverse | GCTTGGAGTTAGGGGACACC |
| CD14 forward | ACGCCAGAACCTTGTGAGC |
| CD14 reverse | GCATGGATCTCCACCTCTACTG |
| DDX5 forward | ATGTCGGGTTATTCGAGTGACC |
| DDX5 reverse | TGTGCGCCTAGCCAAATCAG |
| CDC6 forward | CCAGGCACAGGCTACAATCAG |
| CDC6 reverse | AACAGGTTACGGTTTGGACATT |
| KLK3 forward | GTGTGTGGACCTCCATGTTATT |
| KLK3 reverse | CCACTCACCTTTCCCCTCAAG |
| FOXP3 forward | GTGGCCCGGATGTGAGAAG |
| FOXP3 reverse | GGAGCCCTTGTCGGATGATG |
| BGLAP forward | CACTCCTCGCCCTATTGGC |
| BGLAP reverse | CCCTCCTGCTTGGACACAAAG |
| VDR forward | GTGGACATCGGCATGATGAAG |
| VDR reverse | GGTCGTAGGTCTTATGGTGGG |
| AR forward | CCAGGGACCATGTTTTGCC |
| AR reverse | CGAAGACGACAAGATGGACAA |
| RXR forward | ATGGACACCAAACATTTCCTGC |
| RXR reverse | GGGAGCTGATGACCGAGAAAG |
| RAR forward | AAGCCCGAGTGCTCTGAGA |
| RAR reverse | TTCGTAGTGTATTTGCCCAGC |
| *For cloning* | |
| IGFBP-3 forward | cgggatccATGCAGCGGGCGCGACCCACGCTCTGGGCC |
| IGFBP-3 reverse | cggaattcCTACTTGCTCTGCATGCTGTAGCAGTGCACG |
| *For shRNA* | |
| shIGFBP-3 top strand | ccgg GCTGGTGTGTGGATAAGTA ctcgag TACTTATCCACACACCAGC tttttg |
| shIGFBP-3 bottom strand | aattcaaaaaGCTGGTGTGTGGATAAGTActcgagTACTTATCCACACACCAGC |
| shCYP24A1 top strand | ccggGCATGAAGTTGGGTTCCTTTGctcgagCAAAGGAACCCAACTTCATGCtttttg |
| shCYP24A1bottom strand | aattcaaaaaGCATGAAGTTGGGTTCCTTTGctcgagCAAAGGAACCCAACTTCATGC |
| shCtrl top strand | ccggGCAAGCTGACCCTGAAGTTCATctcgagATGAACTTCAGGGTCAGCTTGCtttttg |
| shCtrl bottom strand | aattcaaaaaGCAAGCTGACCCTGAAGTTCATctcgagATGAACTTCAGGGTCAGCTTGC |
