# Supplementary figures and images for: Molecular evidence of IGFBP-3 dependent and independent VD3 action and its nonlinear response on IGFBP-3 induction in prostate cancer cells
Source: BMC Cancer. 2020 Aug 24;20:802. doi: 10.1186/s12885-020-07310-5 (PMC7446217; doi:10.1186/s12885-020-07310-5)

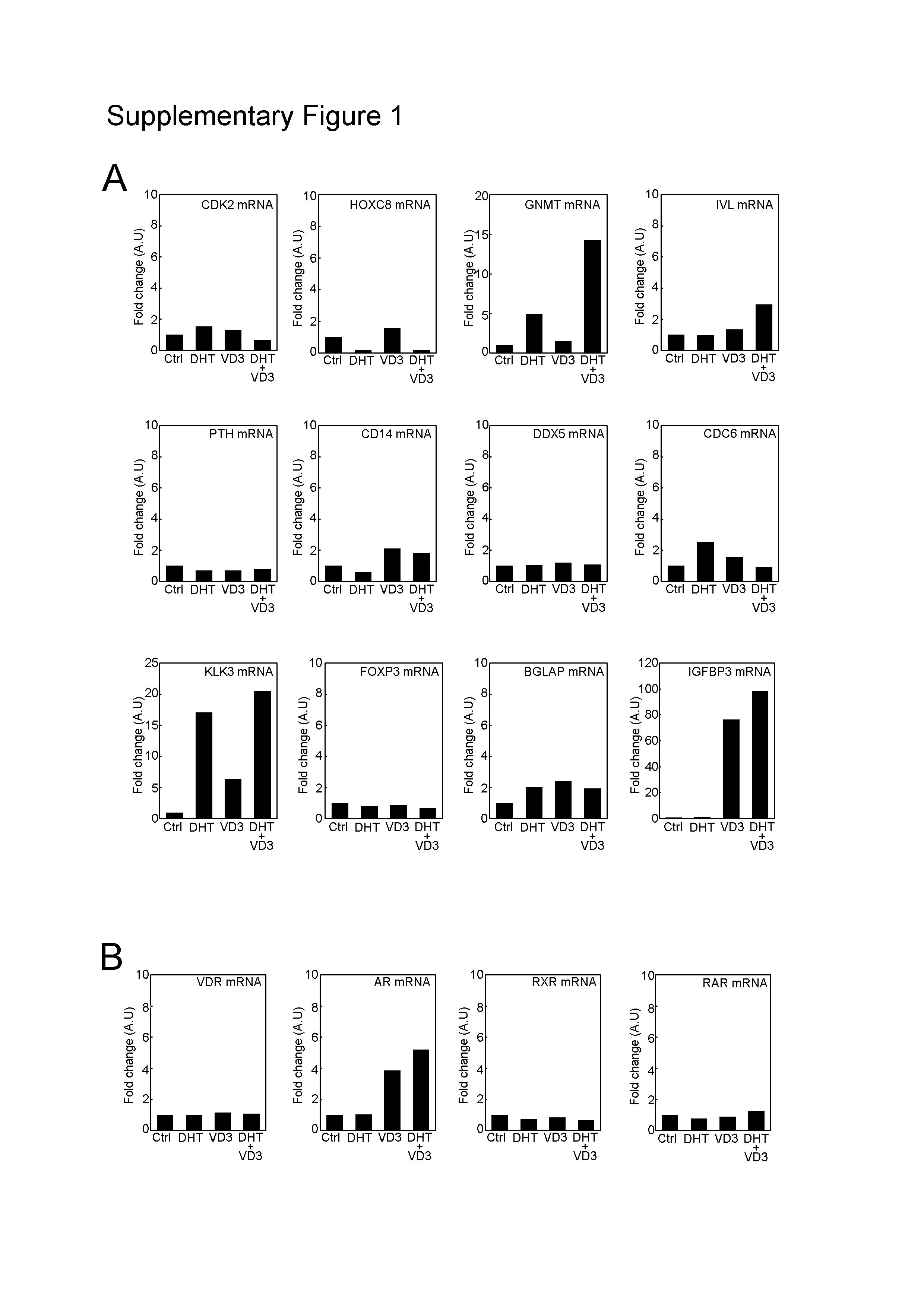

Supplement: Supplementary file 2 — Additional file 2: Supplementary Fig. 1. Expression profiling of the genes induced by VD3 and/or DHT treatment. (a) Expression profiling of the genes related to cellular response that have VDR responsible element on their promoter in LNCaP cells. IGFBP-3 mRNA dramatically responded to VD3 treatment compared to the other genes tested. (b) Expression profiling of the genes related to the VD3 signaling cascade. Androgen receptor (AR), the receptor of DHT, was induced by VD3 treatment. [file 12885_2020_7310_MOESM2_ESM.jpg]

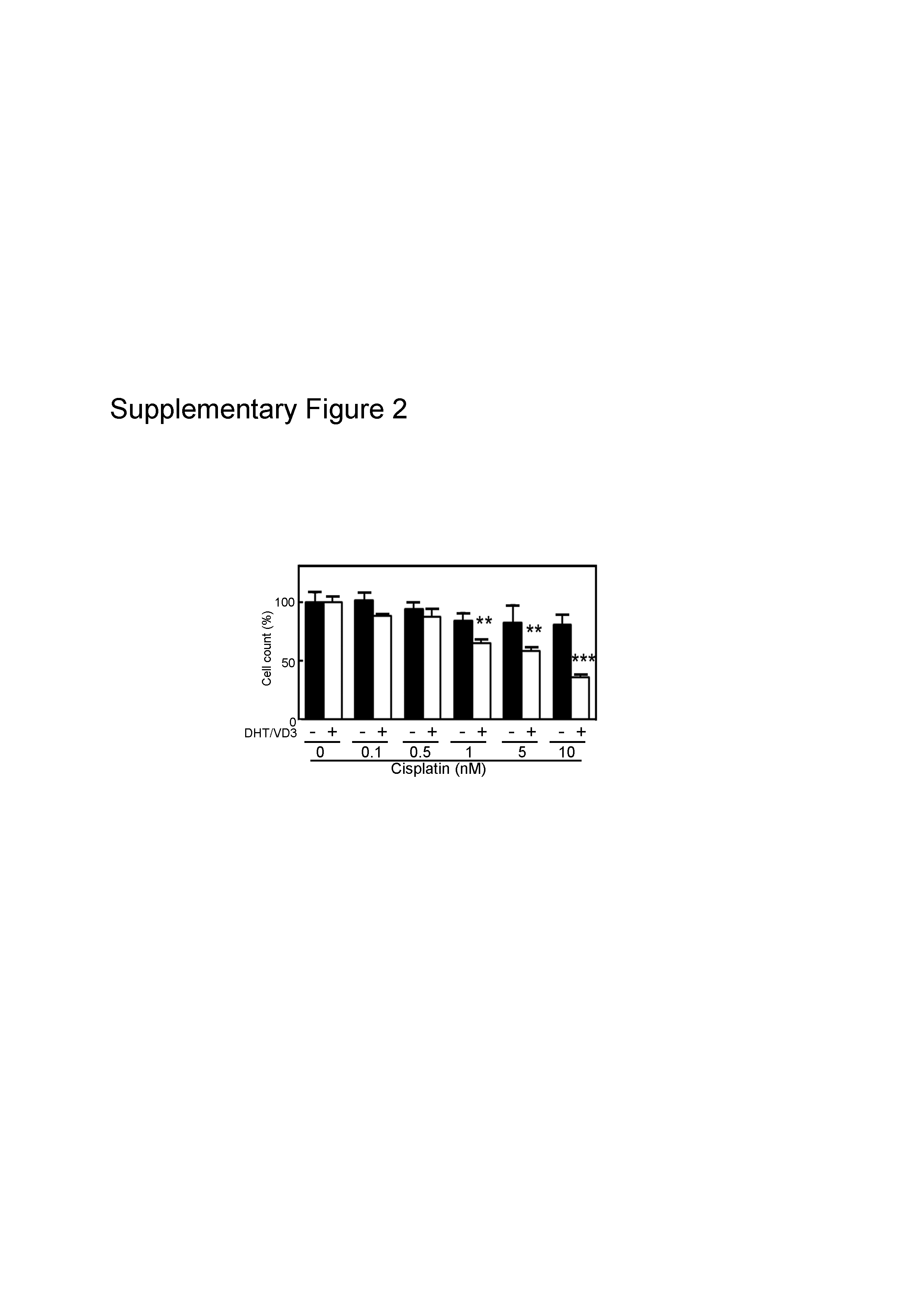

Supplement: Supplementary file 3 — Additional file 3: Supplementary Fig. 2. Cisplatin dose-dependent cytotoxicity in LNCaP cells. LNCaP cells were treated with low-dose cisplatin at the indicated concentration with DHT/VD3. The cells were cultured for 3 days; then, the cell number was measured. At a concentration range of 1–10 nM, DHT/VD3 enhanced the cytotoxicity of cisplatin. [file 12885_2020_7310_MOESM3_ESM.jpg]

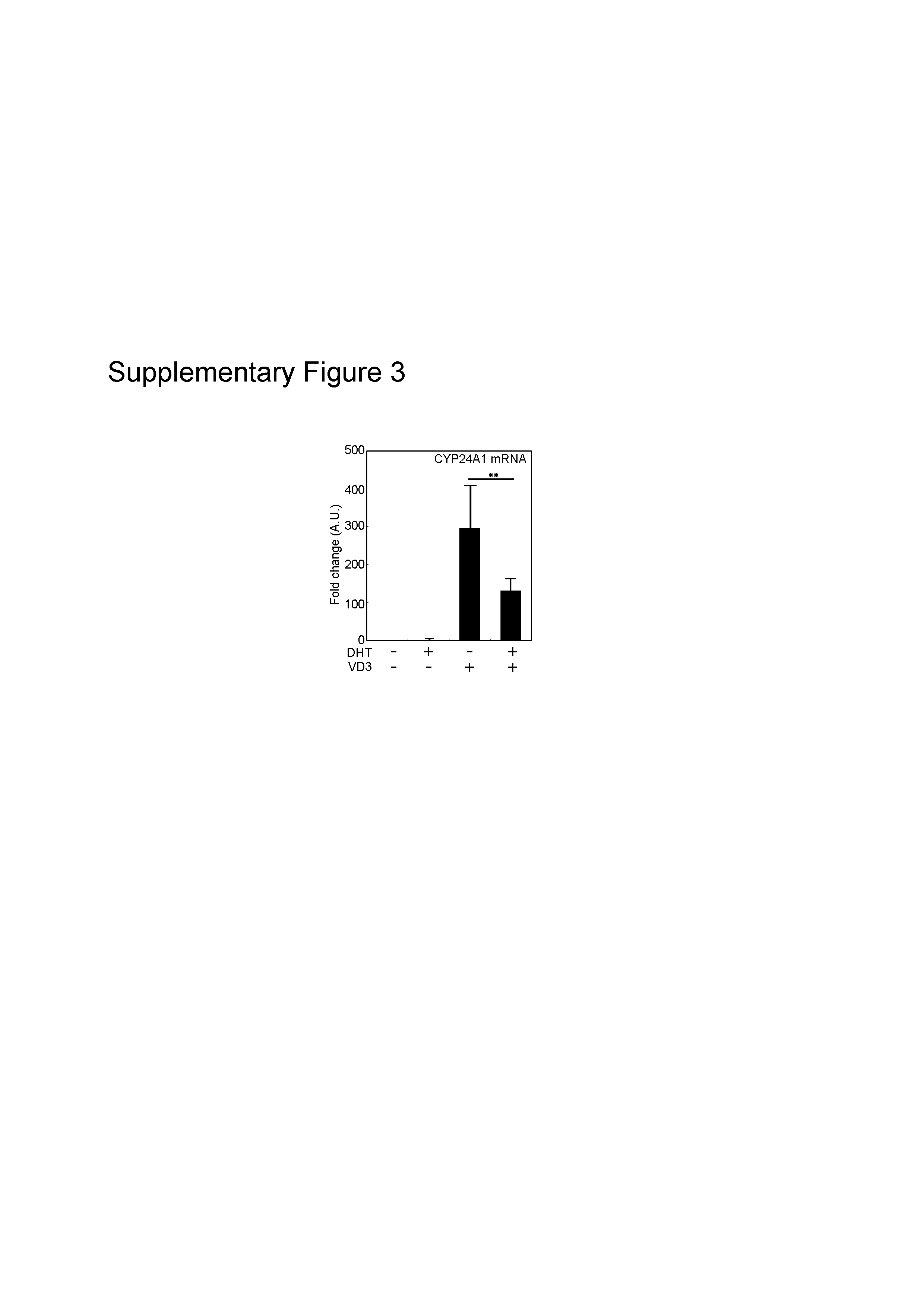

Supplement: Supplementary file 4 — Additional file 4: Supplementary Fig. 3. Establishment of shCYP24A1 expressing LNCaP cells. LNCaP cells were infected with lentivirus that carried shCYP24A1, selected with puromycin, then subjected to VD3 treatment. mRNA of Cyp24a1 was quantitatively observed by qRT-PCR. [file 12885_2020_7310_MOESM4_ESM.jpg]

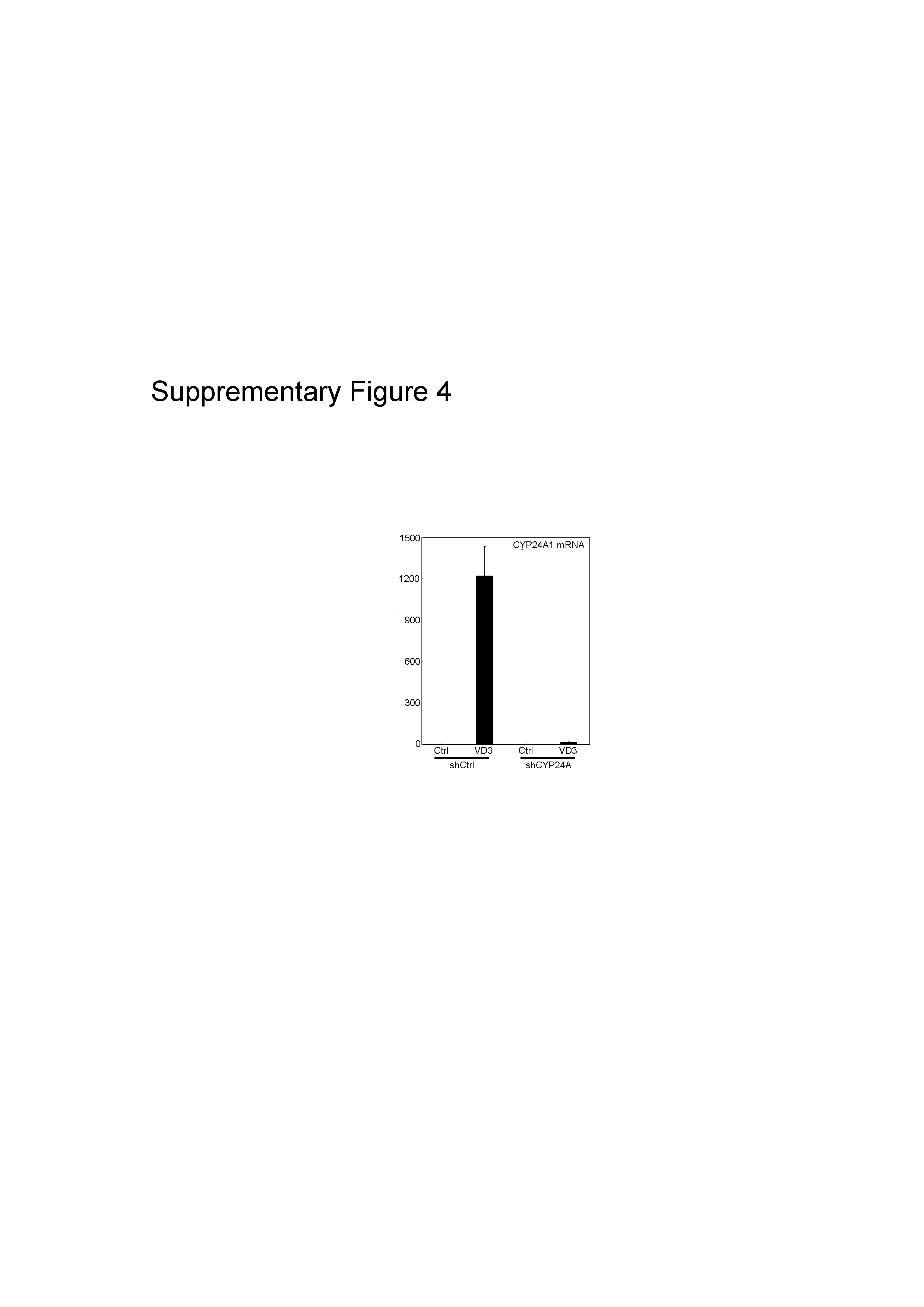

Supplement: Supplementary file 5 — Additional file 5: Supplementary Fig. 4. Induction of the CYP24A1 mRNA expression in LNCaP cells in response to low-dose DHT and VD3 treatment. The expression of CYP24A1 mRNA was induced by low-dose VD3 (10 nM) and was suppressed by simultaneous treatment with DHT (1 nM). [file 12885_2020_7310_MOESM5_ESM.jpg]

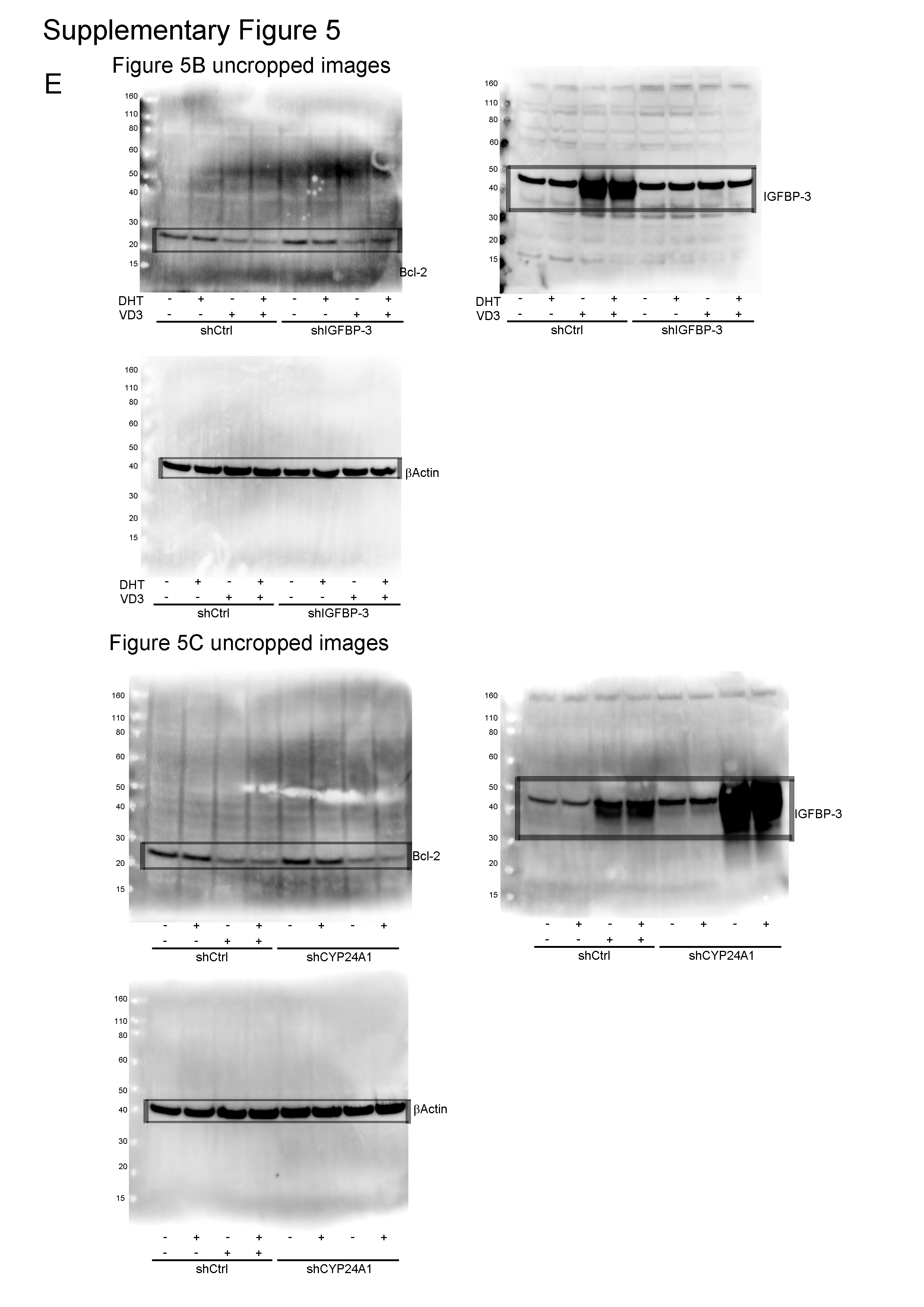

Supplement: Supplementary file 6 — Additional file 6: Supplementary Fig. 5. Original uncropped images of western blot. [file 12885_2020_7310_MOESM6_ESM.zip › Figure S5-4 revised 3ndR3.jpg]

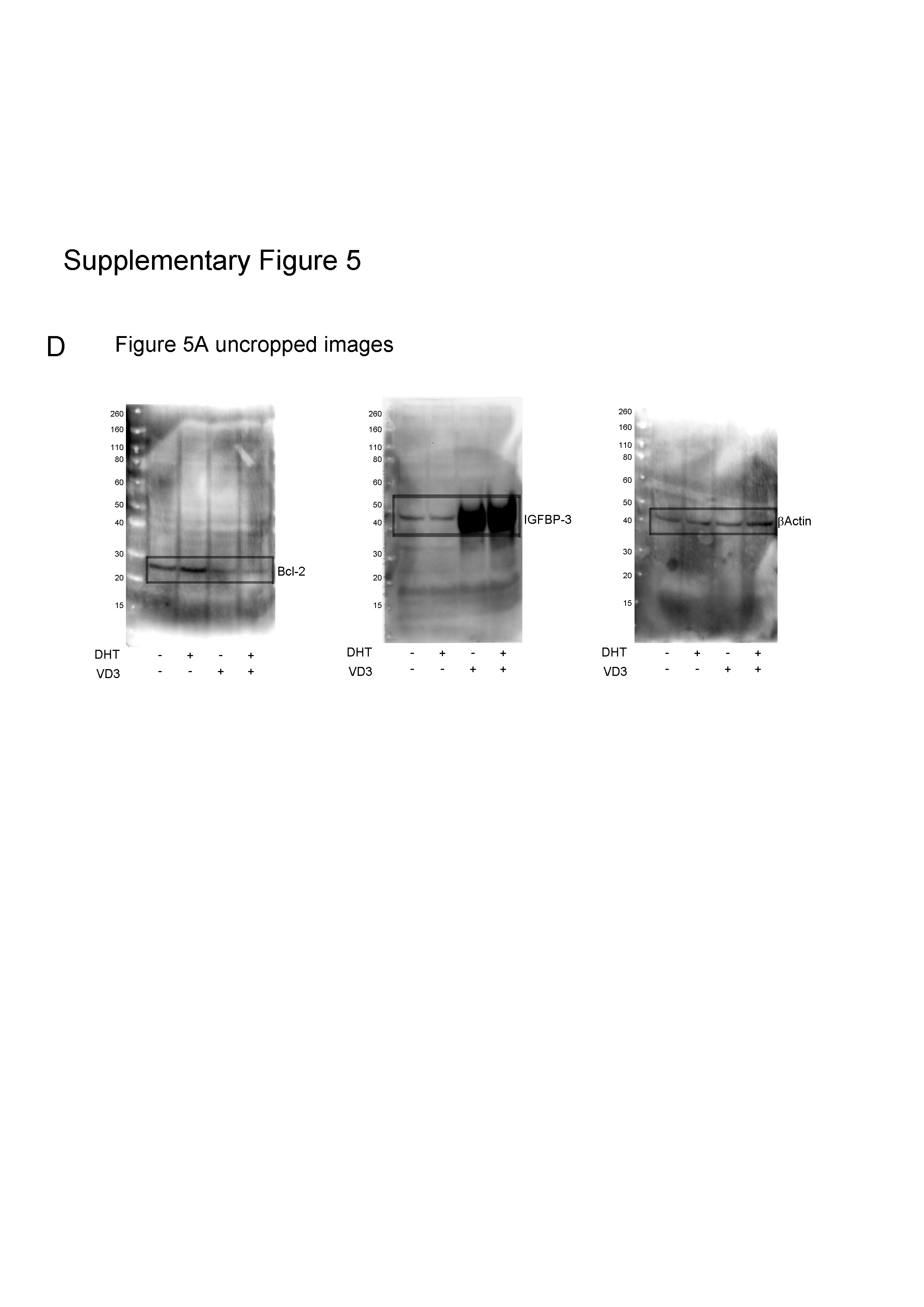

Supplement: Supplementary file 6 — Additional file 6: Supplementary Fig. 5. Original uncropped images of western blot. [file 12885_2020_7310_MOESM6_ESM.zip › Figure S5-3 revised 3ndR3.jpg]

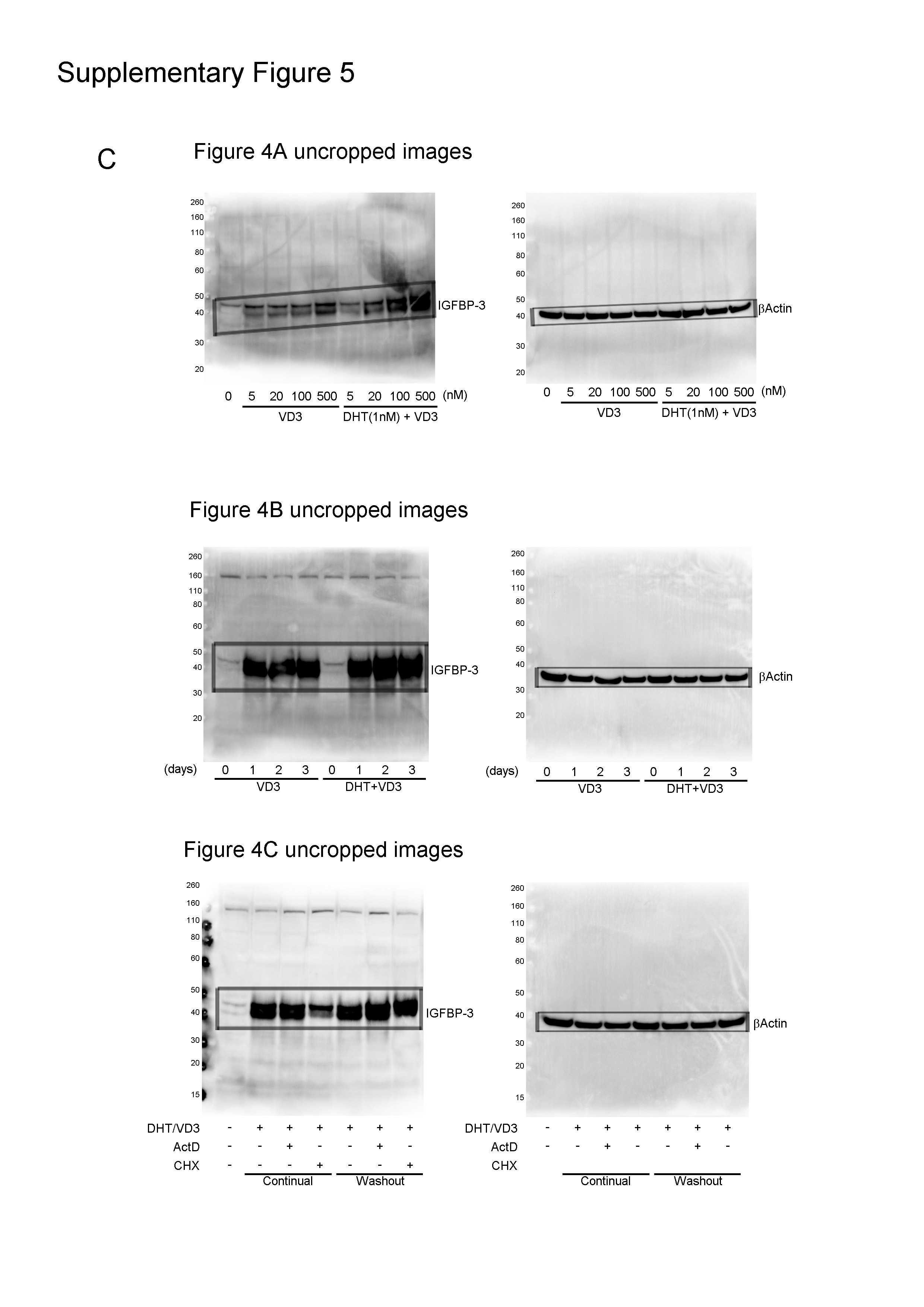

Supplement: Supplementary file 6 — Additional file 6: Supplementary Fig. 5. Original uncropped images of western blot. [file 12885_2020_7310_MOESM6_ESM.zip › Figure S5-2 revised 3rdR3.jpg]

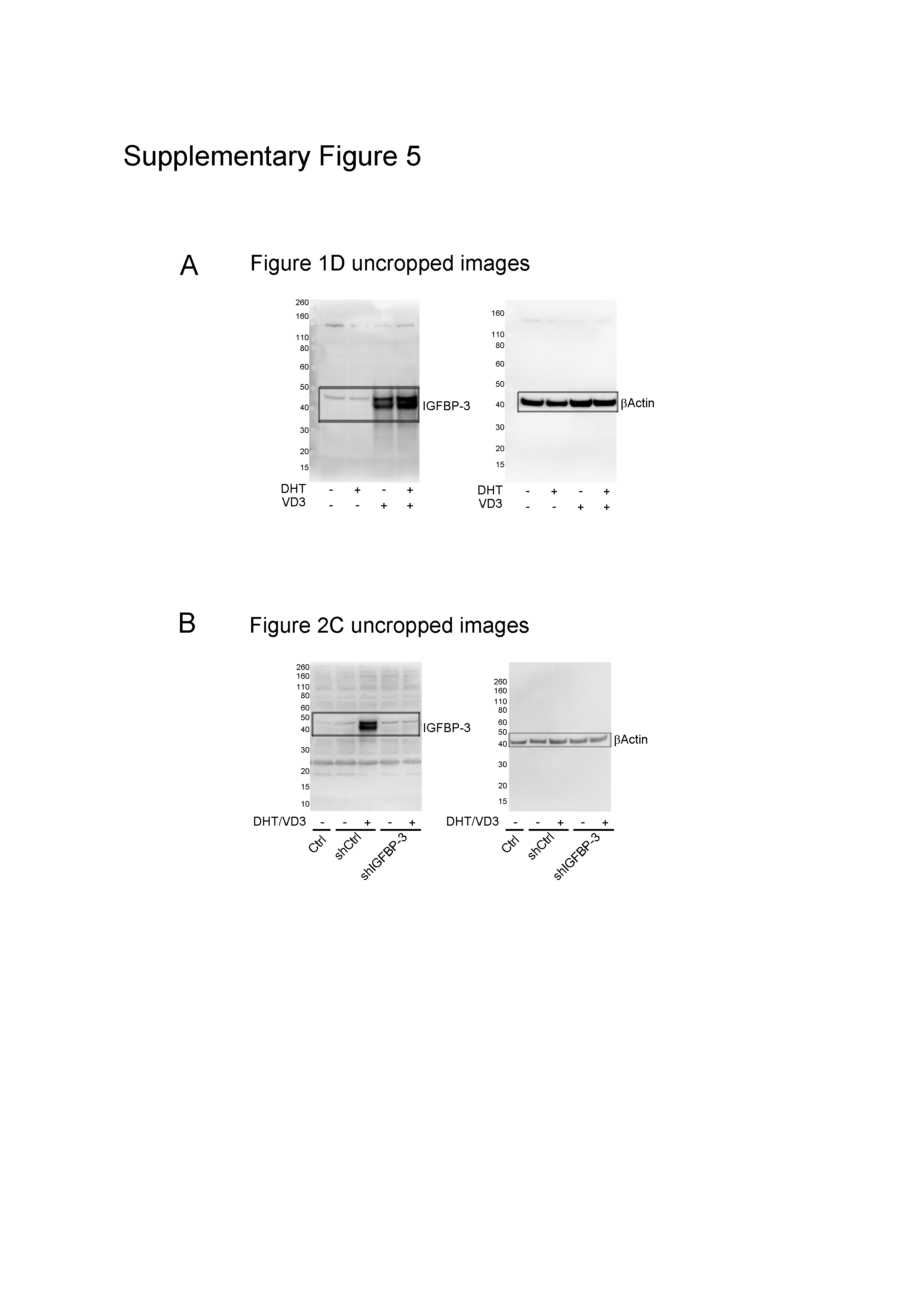

Supplement: Supplementary file 6 — Additional file 6: Supplementary Fig. 5. Original uncropped images of western blot. [file 12885_2020_7310_MOESM6_ESM.zip › Figure S5-1 revised 3rdR3.jpg]
